# Germline Variants of Prostate Cancer in Japanese Families

**DOI:** 10.1371/journal.pone.0164233

**Published:** 2016-10-04

**Authors:** Takahide Hayano, Hiroshi Matsui, Hirofumi Nakaoka, Nobuaki Ohtake, Kazuyoshi Hosomichi, Kazuhiro Suzuki, Ituro Inoue

**Affiliations:** 1 Division of Human Genetics, National Institute of Genetics, Mishima, Japan; 2 Department of Urology, Gunma University Graduate School of Medicine, Maebashi, Japan; 3 Department of Bioinformatics and Genomics, Graduate School of Medical Sciences, Kanazawa University, Ishikawa, Japan; CNR, ITALY

## Abstract

Prostate cancer (PC) is the second most common cancer in men. Family history is the major risk factor for PC. Only two susceptibility genes were identified in PC, *BRCA2* and *HOXB13*. A comprehensive search of germline variants for patients with PC has not been reported in Japanese families. In this study, we conducted exome sequencing followed by Sanger sequencing to explore responsible germline variants in 140 Japanese patients with PC from 66 families. In addition to known susceptibility genes, *BRCA2* and *HOXB13*, we identified *TRRAP* variants in a mutually exclusive manner in seven large PC families (three or four patients per family). We also found shared variants of *BRCA2*, *HOXB13*, and *TRRAP* from 59 additional small PC families (two patients per family). We identified two deleterious *HOXB13* variants (F127C and G132E). Further exploration of the shared variants in rest of the families revealed deleterious variants of the so-called cancer genes (*ATP1A1*, *BRIP1*, *FANCA*, *FGFR3*, *FLT3*, *HOXD11*, *MUTYH*, *PDGFRA*, *SMARCA4*, and *TCF3*). The germline variant profile provides a new insight to clarify the genetic etiology and heterogeneity of PC among Japanese men.

## Introduction

Prostate cancer (PC) is the second most common cancer (1.1 million new cases in 2012) in men worldwide and had the highest incidence rate in developed countries [1]. Family history is one of the established risk factors in addition to age and population [2]. A meta-analysis showed that first-degree relatives of an affected patient are 2.48 times more likely to develop PC [3]. The study of the Nordic Twin Study of Cancer cohort indicated that heritable factors may be attributable to 58% of the PC risk and liability [4]. In addition to family and twin studies, differences of the PC incidence in different populations suggest that genetic components and life-style are important factors in PC risk [1, 2, 5].

Genome-wide association studies and meta-analyses identified 99 variants associated with PC risk [2, 6, 7]. These variants explain 33% of the familial risk of PC in European descendants [7]. Only two susceptibility genes for PC, *BRCA2* and *HOXB13*, were identified [2]. Germline variants of *BRCA2* are established risk factors of PC [8]. *HOXB13* is a key transcription factor for PC and the association between a rare variant of *HOXB13* G84E and PC risk was confirmed in a recent large-scale study [9]. To date, the *HOXB13* G84E variant has been found only among European descendants. Identification of different *HOXB13* variants in individuals with non-European descent, including *HOXB13* G135E in Chinese men, indicates allelic heterogeneity of *HOXB13* variants depending on populations [10, 11, 12].

The age-standardized incidence rate (ASIR) among Japanese men was relatively low compared with that among European men [1, 5]. However, ASIR has been increasing in Japan probably owing to lifestyle changes [1, 13]. Genetic exploration of PC among Japanese men is important to understand the development of PC at the population level. In our previous study, we identified PC susceptibility loci of chromosomes 8p23 and 1p36 in Japanese patients with affected siblings by genome-wide linkage analysis. However, the confirmative linkage results were not obtained [14]. Comprehensive study for germline variants of Japanese patients with PC has not been reported.

To find novel responsible genes for PC, exome sequencing (exome-seq) of 81 patients with PC from seven large families (three or four patients per family) and 59 small families (two patients per family) was conducted.

## Materials and Methods

### Ethics Statement

The study protocols were approved by the Institutional Review Boards of Gunma University (No. 5, 2013.12.2) and National Institute of Genetics (No. 26–6, 2014.8.8). Each participant provided written informed consent for the collection of samples and subsequent analyses.

### PC families and study design

Sixty-six families were categorized as large PC families and small PC families: The large PC families consisted of three or four patients with PC (22 patients in seven families). The small PC families are pairs of patients with PC (118 patients in 59 families). Only patients were recruited. All of the 22 patients of the large families and probands of small families were analyzed by exome-seq. Shared variants in the patients of small families were confirmed by Sanger-seq using the counterpart of PC pairs in each family.

### Clinical information

All 140 patients with PC were histologically diagnosed at Gunma university hospital and its affiliated hospitals. Patients had a mean age at diagnosis of 69.0 (range, 40–88 years). Gleason scores [15] were lower than 7 in 42 patients and equal to 7 or higher in 97 patients (unknown in one patient).

### DNA preparation and exome-seq

Genomic DNA was isolated from peripheral blood using a GENOMIX kit (Talent srl. Treisete, Italy). Fragmentation and adaptor tagmentation of the genomic DNA followed by hybridization for capturing probes were performed using a SureSelect Human All Exon V5+lncRNA (Agilent) for preparing capture libraries. The libraries were sequenced using the Illumina HiSeq 2500 (Illumina) with 150 base-paired end modules (for the large PC families) or 100 base-paired end modules (for the small PC families).

### Exome-seq data analyses

Sequencing reads were mapped to a reference genome (hg19) using BWA-mem [16] and SAMtools [17]. Picard MarkDuplicatesWithMateCigar module (http://broadinstitute.github.io/picard/) was used for removing duplicate reads. Local realignment of reads around known indels and recalibration of base quality were performed using Genome Analysis Toolkit (GATK) IndelRealigner and BaseRecalibrator module, respectively [18, 19, 20]. Variant call and genotyping were performed using GATK HaplotypeCaller. Vcf files were decomposed and normalized by vt program [21]. For the large families, variant calls in the same family were combined with GATK CombineGVCF module. Shared variants in same family members were extracted using snpEff and SnpSift [22].

### Filtering and prioritizing of variants

The variants_reduction.pl script of ANNOVAR was used for filtering [23]. We focused on exonic and splicing variants. The synonymous variants were filtered out. Variants in the genomic super duplicated regions were removed. Database-registered single nucleotide polymorphisms (SNPs) were removed, except for clinically reported variants using dbSNP Flagged information. Rare variants with minor allele frequency (MAF) of <0.001 were filtered in from information from the NHLBI GO Exome Sequencing Project [24] and the 1000 Genomes Project (1KGP) [25]. The remaining variants were annotated using table_annovar.pl script of ANNOVAR. For prioritization, the hiPIVE module in Exomiser was used [26, 27], which compiled information from phenotype (human, mouse, and zebra fish), protein–protein interaction, and in silico predictions (SIFT, Polyphen2, and MutationTaster). The OMIM term (OMIM: 176807) and HPO term (HPO: 0012125) for “Prostate Cancer” were used for the phenotype input for the hiPIVE module. Combined score greater than or equal to 0.80 of Exomiser was set as the threshold. Overlapped genes from ANNOVAR and Exomiser results were extracted as candidate genes.

### Allele frequency databases

Allele frequency data from more than 60,000 individuals of Exome Aggregation Consortium (ExAC) was used as reference (http://exac.broadinstitute.org/). For allele frequencies in Japanese, we referred to two databases of the integrative Japanese Genome Variation Database (iJGVD) [28, 29] and the Human Genetic Variation Database (HGVD) [30]. The iJGVD was derived from more than 1,000 healthy Japanese individuals using whole genome sequencing. Data were downloaded on 26 April 2016. The HGVD collected exome variants information from more than 1,000 Japanese individuals. Data release version 1.42 was used.

### The CGC genes

To further extract cancer-related genes, information from the CGC database [31] was used. CGC is a census of cancer genes with variants obtained from literature searches. A gene list of CGC was downloaded on 29 October 2015 from the web site (http://cancer.sanger.ac.uk/census/).

### Functional prediction of variants and selection of genes for Sanger-seq

For the functional prediction of variants, we referred to two in silico prediction scores of LR and RadialSVM, which are ensemble scores from nine prediction methods (FATHMM, LRT, MutationAssessor, MutationTaster, PolyPhen-2, SIFT, GERP++, PhyloP, and SiPhy) and allele frequency [32]. These two ensemble scores are implemented in the ANNOVAR [23].

Variants of the CGC genes predicted from both of the LR and RadialSVM were analyzed by Sanger-seq.

## Results

### Results of exome-seq

**Fig 1:**
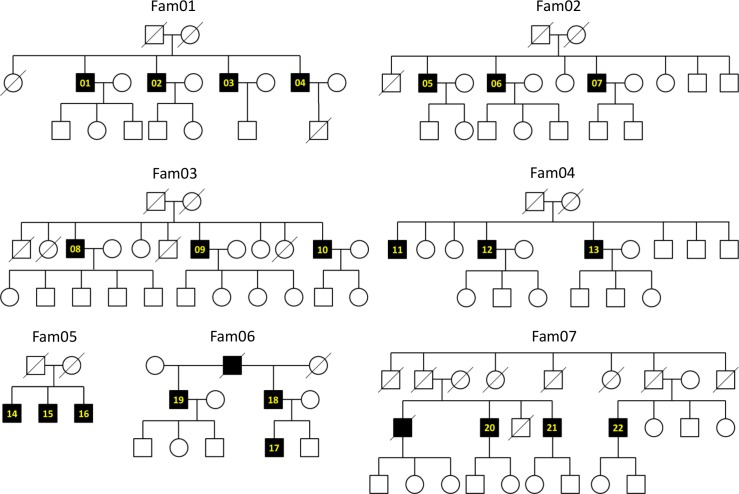
Pedigrees of the seven large PC families. Solid black rectangles represent affected patients with PC. Patients with PC analyzed by exome-seq were numbered (from 01 to 22). PC, prostate cancer.

Germline variants were identified in 81 patients with PC from 66 families through exome-seq data analyses. Twenty-two and fifty-nine patients were from 7 large PC families (Fig 1) and 59 small PC families, respectively. Mapping results achieved the average read depth of 97 and 72, and the coverage of 95.2% and 80.7% at read depth of 20 for the large families and small families, respectively (S1 and S2 Tables). A total of 1,082,617 variants (154,659 per family) were detected and an average of 83,197 variants (from 79,146 to 87,027) was shared in each large family (S3 Table). In the small families, an average of 116,536 variants (from 113,178 to 119,238) was detected (S4 Table). After filtering and prioritizing variants (Materials and Methods), 564 genes with variants remained. In these genes with variants, none of the genes from the 66 families had a frequency greater than 12%, except for *MUC6* (66 of 66, 100%), *TBP* (44 of 66, 66.7%), and *MUC5B* (32 of 66, 48.5%). We excluded mucin genes (*MUC6* and *MUC5B*) because variants of these genes were frequently detected in public exome data sets and less likely to be associated with diseases [33]. After removing common variants of *TBP* observed in HGVD (MAF > 0.3), the frequency of *TBP* was lower than 12% (3 of 66, 4.5%).

**Fig 2:**
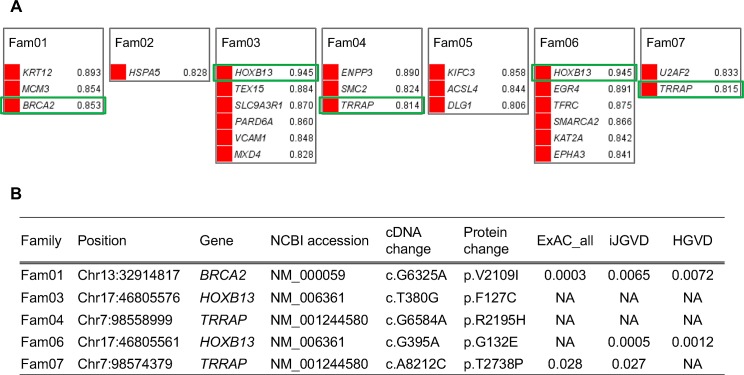
Shared genes with variants in the large PC families. (A) Twenty-two genes in the seven large families remained after filtering and prioritizing. Known susceptibility genes (*BRCA2* and *HOXB13*) and one novel gene (*TRRAP*) are shown by green rectangles. The combined scores of Exomiser are shown on the right side of the gene names. (B) Variant status of *BRCA2*, *HOXB13*, and *TRRAP*. ExAC_all, MAF of all subjects in the ExAC; iJGVD, MAF in the iJGVD; HGVD, MAF in the HGVD; NA, Not applicable. PC, prostate cancer.

To find shared genes with variants, we first focused on the seven large PC families with more than three patients. In the large families, *TRRAP* was identified in two families (Fam04 and Fam07) together with known PC susceptibility genes of *BRCA2* (Fam01) and *HOXB13* (Fam03 and Fam06) in a mutually exclusive manner (Fig 2A). The *BRCA2* variant (V2109I) was found in a patient with breast cancer (BC) with family history of pancreas cancer [34] and a patient with esophageal cancer with family history of gastric cardia cancer [35]. For two *HOXB13* variants (F127C and G132E), the allele frequency information was not reported in the ExAC database. Although we found allele frequency information for *HOXB13* G132E in Japanese databases (MAF = 0.00048 in iJGVD and MAF = 0.0012 in HGVD), this might not reject the pathogenicity of the variants considering the frequency of PC. The *TRRAP* R2195H variant was a novel indicating pathogenic and the *TRRAP* T2738P variant was found in the ExAC (MAF = 0.028) and iJGVD (MAF = 0.027) databases; therefore, the pathogenicity of the variant would be reserved (Fig 2B). All variants were heterozygous in the families.

### Shared variants in the small PC families

**Fig 3:**
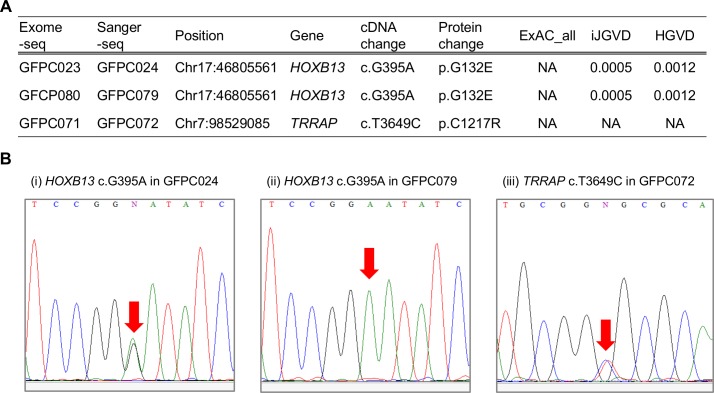
Variants of *HOXB13* and *TRRAP* in the 59 small PC families. (A) Variant status of *HOXB13* and *TRRAP*. ExAC_all, MAF of all subjects in the ExAC; iJGVD, MAF in the iJGVD; HGVD, MAF in the HGVD; NA, Not applicable. (B) Results of Sanger-seq for shared variants of *HOXB13* and *TRRAP* (i) Heterozygous variant of *HOXB13* G132E (c.G395A) in GFPC024. (ii) Homozygous variant of *HOXB13* G132E (c.G395A) in GFPC079. (iii) Heterozygous variant of *TRRAP* C1217R (c.T3649C) in GFPC072. The positions of variants are indicated by red arrows. PC, prostate cancer.

We further examined shared variants of *BRCA2*, *HOXB13*, and *TRRAP* in the 59 small families of PC pairs. A variant of *BRCA2* R18H was shared in a family (GFPC043/044). However, this variant is reported as “Benign” in the ClinVar database [36]; thus, it was excluded from candidate variants. Five *BRCA2* variants (L61P, H1458R, G2508S, H3056Y, and R3384X) detected in five probands were not shared in the counterpart of each family. The same variant of *HOXB13* G132E, which was found in a large family (Fam06), was also shared in two small families (GFPC023/024 and GFPC079/080). We also identified a shared novel variant of *TRRAP* C1217R indicating pathogenicity in a family (GFPC071/072) (Fig 3). All shared variants were heterozygous except for *HOXB13* G132E in GFPC079 (homozygous).

**Fig 4:**
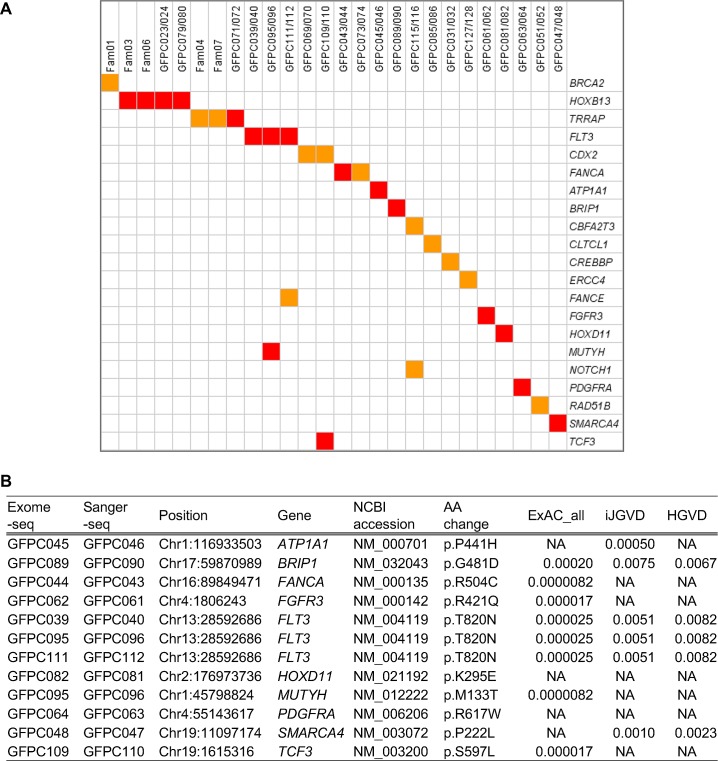
Shared genes with variants. (A) Heat map of the shared genes with variants. Each column shows the family identification of large PC families or PC pairs of the small PC families. Each row shows the gene names and shared variants are filled with red (deleterious) or orange (nondeleterious) color. (B) Deleterious variants of the Cancer Gene Census genes. The variant status is shown. ExAC_all, MAF of all subjects in the ExAC; iJGVD, MAF in the iJGVD; HGVD, MAF in the HGVD; NA, Not applicable; PC, prostate cancer.

In the remaining 56 small families without shared variants of *HOXB13* and *TRRAP*, patients with PC from 44 families had at least one variant of cancer genes of the CGC (Materials and Methods). In a total of 74 variants in 60 CGC genes, we performed Sanger-seq for 50 variants in 46 genes and confirmed shared variants of 18 genes (Fig 4 and S5 Table). Ten variants in 10 genes (*ATP1A1*, *BRIP1*, *FANCA*, *FGFR3*, *FLT3*, *HOXD11*, *MUTYH*, *PDGFRA*, *SMARCA4*, and *TCF3*) were predicted to be deleterious by two prediction methods, RadialSVM and LR. Notably, the same variant of *FLT3* T820N was found in four probands (GFPC039, GFPC095, GFPC098, and GFPC111) and shared in three families (GFPC039/40, GFPC095/096, and GFPC111/112).

### Clinical relevance

**Fig 5:**
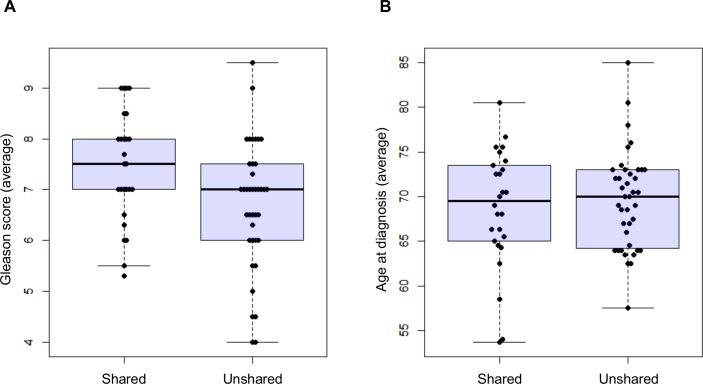
Associations between clinical features and shared variant status. (A) Comparison of Gleason score between the shared families and unshared families. Gleason scores were averaged in each family. One family lacking Gleason score was omitted. (B) Comparison of age at diagnosis between the shared families and unshared families. Ages at diagnosis were averaged in each family. Shared, families with shared variants; unshared, families with unshared variants.

In total, 58 patients from 26 families shared variants of *BRCA2*, *HOXB13*, *TRRAP*, or CGC genes (Fig 4A). We assessed associations between clinical features (Gleason score and age at diagnosis) and shared variant status. We compared the families with shared variants (shared families) and without shared variants (unshared families). We found that the shared families showed high Gleason scores (averaged scores in each family) compared with unshared families (Wilcoxon rank sum test p value = 0.027) (Fig 5A). The mean age at diagnosis of the shared families and unshared families was 68.7 (range from 53.7 to 80.5 years) and 69.6 (range from 57.5 to 85.0 years), respectively, showing no statistical difference (Wilcoxon rank sum test p value = 0.99) (Fig 5B).

## Discussion

In this study, we profiled germline variants in a large numbers of patients with PC in Japanese families. In the total of 140 patients with PC from 66 families, *TRRAP* was identified as a novel candidate gene for PC together with known susceptibility genes of *BRCA2* and *HOXB13*.

TRRAP is a common component of many histone acetyltransferase (HAT) complexes, and it is involved in transcriptional regulation and DNA repair [37]. Protein expression of TRRAP was low in BC compared with matched normal breast tissues, indicating a tumor suppressive role of TRRAP for BC [38]. The association between germline variants of *TRRAP* and PC remains unclear, and further studies are needed to clarify this association.

Two variants of *HOXB13* (F127C and G132E) were identified. Both of the variants were considered to be deleterious and were located at near the homeobox protein Hox1A3 N-terminal domain (residues 21–123) [12]. We could not find the specific European *HOXB13* G84E variant among Japanese samples, which was consistent with previous reports [10, 11, 12]. A large-scale study for *HOXB13* variants in a Chinese population (96 patients with PC) failed to detect the two *HOXB13* variants that were detected in the present study, indicating the allelic heterogeneity of *HOXB13* variants in Asian populations.

The variant of *BRCA2* V2019I was found in patients with family history of cancer [34, 35]. This variant was also found in a hereditary breast and ovarian cancer study in Japanese women. Further, this variant was reported as a variant of uncertain significance [39]. Further evidence may be required to determine the pathogenicity of the variant.

We further identified shared variants of the CGC genes in the remaining small families, and 10 variants were considered to be deleterious (Fig 4 and S5 Table). Three genes (*BRIP1*, *FANCA*, and *MUTYH*) were DNA repair genes. Additionally, rare germline variants of *BRIP1* and *MUTYH* were previously identified in PC [40]. A deleterious germline mutation of *FANCA* was found in familial BC [41]. Three receptor tyrosine kinase (RTK) genes (*FGFR3*, *FLT3*, and *PDGFRA*) were identified. Somatic variants of *RTK* have been reported to confer oncogenic function in various cancers and inhibitors of RTK are targeted drugs for the anticancer therapy [42]. Germline variants of *FGFR3* and *PDGFRA* were also found in medulloblastoma and gastrointestinal stromal tumors, respectively [43, 44]. The deleterious variant of *FLT3* T820N in three families located on the C lobe of the kinase domain [45] could affect kinase activity. The patient (GFPC097) who did not share the *FLT3* T820N variant could be a sporadic case. *HOXD11* and *TCF3* are coding transcription factors. Somatic fusion genes of these genes such as *NUP98-HOXD11* and *TCF3-HLF* were found in leukemia [46, 47]. Frequent germline and somatic mutations of *SMARCA4* in small cell carcinoma of the ovary of hyper calcemic type (SCCOHT) suggested a tumor suppressor role [48, 49]. Somatic variants of *ATP1A1* were reported to cause aldosterone producing adenomas in primary aldosteronism [50]. An association between germline mutations of four genes (*FLT3*, *HOXD11*, *TCF3*, and *ATP1A1*) and cancer has not been reported.

We consider that the main limitation of this study was that our study design lacked the inclusion of healthy controls. Additionally, the heterogeneity of PC causality, which is shown here, limits the current causal variant detection in the families studied. Further association studies using case and control samples and functional studies are required to evaluate the contribution of these variants in the development of PC.

In this study, we re-evaluated a collection of familial patients with PC in our previous genome-wide linkage study [14] using exome analysis. High Gleason scores in the shared families compared with unshared families suggested that germline variants of the candidate genes could contribute to the malignancy of PC. Our results provide a resource for further understanding of PC development at population level.

## Supporting Information

S1 Table Depth and coverage results of exome-seq in the large prostate cancer families. Family, ID of the large prostate cancer families; ID, ID of each individual; Depth, mean depth of each sample; Coverage, coverage at depth of 20.
(XLSX)

S2 Table Depth and coverage results of exome-seq in the small prostate cancer families. ID, ID of each individual; Depth, mean depth of each sample; Coverage, coverage at depth of 20.
(XLSX)

S3 Table Numbers of variant in the seven large prostate cancer families. Family, ID of the large prostate cancer families; Variants; total number of variants detected in each family; Shared variants, number of shared variants in each family.
(XLSX)

S4 Table Numbers of variant in the 59 small prostate cancer families. ID, ID of each individual; Variants, number of variants detected in each individual.
(XLSX)

S5 Table Variants of the Cancer Gene Census genes. This table is an output of ANNOVAR. Additional columns of “Sanger-seq” and “SangerResutls” were added. Done, Sanger-seq was performed; Undone, Sanger-seq was not performed; Shared, shared variant was confirmed; WT, wild type; Not confirmed, variant status was not confirmed.
(XLSX)
